# The Association between Symptoms, Pain Coping Strategies, and Physical Activity Among People with Symptomatic Knee and Hip Osteoarthritis

**DOI:** 10.3389/fpsyg.2012.00326

**Published:** 2012-09-03

**Authors:** Susan L. Murphy, Anna L. Kratz, David A. Williams, Michael E. Geisser

**Affiliations:** ^1^Department of Physical Medicine and Rehabilitation, University of MichiganAnn Arbor, MI, USA; ^2^Geriatric Research, Education, and Clinical Center, VA Ann Arbor Health Care SystemAnn Arbor, MI, USA; ^3^Chronic Pain and Fatigue Research Center, Department of Anesthesiology, University of MichiganAnn Arbor, MI, USA; ^4^Division of Rheumatology, Department of Internal Medicine, University of MichiganAnn Arbor, MI, USA; ^5^Department of Psychiatry, University of MichiganAnn Arbor, MI, USA; ^6^Department of Psychology, University of MichiganAnn Arbor, MI, USA

**Keywords:** accelerometry, chronic pain, coping strategies, ecological momentary assessment, physical activity

## Abstract

Effective use of coping strategies by people with chronic pain conditions is associated with better functioning and adjustment to chronic disease. Although the effects of coping on pain have been well studied, less is known about how specific coping strategies relate to actual physical activity patterns in daily life. The purpose of this study was to evaluate how different coping strategies relate to symptoms and physical activity patterns in a sample of adults with knee and hip osteoarthritis (OA; *N* = 44). Physical activity was assessed by wrist-worn accelerometry; coping strategy use was assessed by the Chronic Pain Coping Inventory. We hypothesized that the use of coping strategies that reflect approach behaviors (e.g., Task Persistence), would be associated with higher average levels of physical activity, whereas avoidance coping behaviors (e.g., Resting, Asking for Assistance, Guarding) and Pacing would be associated with lower average levels of physical activity. We also evaluated whether coping strategies moderated the association between momentary symptoms (pain and fatigue) and activity. We hypothesized that higher levels of approach coping would be associated with a *weaker* association between symptoms and activity compared to lower levels of this type of coping. Multilevel modeling was used to analyze the momentary association between coping and physical activity. We found that higher body mass index, fatigue, and the use of Guarding were significantly related to lower activity levels, whereas Asking for Assistance was significantly related to higher activity levels. Only Resting moderated the association between pain and activity. Guarding, Resting, Task Persistence, and Pacing moderated the association between fatigue and activity. This study provides an initial understanding of how people with OA cope with symptoms as they engage in daily life activities using ecological momentary assessment and objective physical activity measurement.

## Introduction

Osteoarthritis (OA) is a chronic condition that affects 27 million people in the United States and is a leading cause of disability in adults. For people with knee and hip OA, the experience of symptoms can greatly impair quality of life. Pain is the main reason people seek treatment. Pain in OA affects the ability to engage in activities of daily living, work, and other meaningful activities (Hill et al., 1999; Boutron et al., 2008; Grotle et al., 2008). Fatigue, although not as well studied in OA, is one of the most frequently reported OA symptoms (Wolfe et al., 1996; Power et al., 2008), and one of the strongest predictors of functional disability (Wolfe, 1999). While a link between symptoms of pain and fatigue has been established with physical disability in OA, less is known about how pain, fatigue, and psychosocial factors, such as coping ability, influence physical activity on a day-to-day basis. A better understanding of these interrelationships offers the potential for insight into more effective ways to manage OA.

According to Lazarus and Folkman (1984), people develop methods of coping in response to stressors such as pain and fatigue. Coping strategies may be cognitive (such as an attribute or belief to assign meaning to a stressful situation) or behavioral (such as an action-oriented response). The development of a strategy to cope with adversity such as pain does not insure that it is adaptive. In addition, strategies may be adaptive in the short-term but prove to be maladaptive longer term if pain becomes chronic (Jensen, 1991; Hasenbring and Verbunt, 2010). There have been efforts to identify and categorize specific coping behaviors into maladaptive and adaptive types (Ersek, 2006; Tan et al., 2011; Englbrecht et al., 2012), with different terminology to describe the categories and often times different scales that have been developed to assess the proposed dimensions. One popular means of categorizing behavioral coping relates to how people engage in or approach activity in the context of their medical symptoms. According to this perspective, behavioral strategies that relate to activity broadly reflect three dimensions: avoidance, persistence, and pacing.

### Avoidance

The fear-avoidance model (Vlaeyen and Linton, 2000) describes how the pain experience can lead to a pathway where habitually avoiding activity promotes a cycle of disuse and disability. In essence, catastrophizing about pain and its potential consequences, or ruminating, feeling helpless, or exaggerating the threat of pain, leads to pain-related fear or anxiety (Norton and Asmundson, 2003), which causes avoidance behaviors and ultimately reinforces this negative cycle (Leeuw, 2007). Consistent with the idea that avoidance behaviors are maladaptive coping strategies, research examining avoidance usually associates these behaviors to disability or other outcomes such as depressed mood or maintenance of pain. In OA, use of rest as a coping strategy has been associated with physical disability in cross-sectional studies (Hopman-Rock et al., 1998; van Baar et al., 1998) as well as in longitudinal studies (Steultjens, 2001). In addition, rest and restricting activities have also been related to negative mood and pain at follow-up in people with OA (Hampson et al., 1996). Another strategy considered to be avoidant, Guarding (e.g., bracing, limping, flinching, stiffening), had the strongest independent association with disability (Tan et al., 2001) in a study of male veterans with chronic pain. In a similar sample, Tan et al. (2011) found that Guarding and Resting were associated with depression and higher levels of pain interference. Further, Guarding and Asking for Assistance (a behavior considered avoidant because other people are solicited to complete tasks) have also been associated with disability in people who have fibromyalgia (Karsdorp and Vlaeyen, 2009).

### Activity persistence

Activity persistence in general refers to persisting in an activity, even in the context of symptoms that may present barriers to engaging in that activity. Persistence may be considered either adaptive or maladaptive, depending on the degree or intensity of activity persistence. For instance, in the avoidance-endurance model of chronic pain, “endurance copers” are those people who persist in activity despite severe pain. They may have high levels of unhealthy activity and may respond to pain by being *excessively* persistent instead of avoidant (Hasenbring and Verbunt, 2010). Studies have revealed both positive and negative relationships between persistence and disability (Jensen, 1991; Kindermans, 2011). Kindermans (2011), who performed a factor analysis using several measures representing the persistence construct, found that excessive persistence (such as doing too much or not respecting one’s physical limits) was positively associated with disability on the Pain Disability Index and with depression. In contrast, task-contingent persistence has been found to be associated with less disability (Jensen, 1991; Jensen et al., 1995).

### Pacing

Time-based activity pacing is a behavioral strategy in which people learn to lessen the effect of symptoms on activity by breaking up activities into smaller pieces, and alternating activity and rest periods to maintain a steady pace (Fordyce, 1976). These behaviors are thought to attenuate the “overactivity-underactivity” cycle in which excessive activity can lead to symptom flares that require a prolonged period of rest to recover (Birkholtz et al., 2004). In some studies, Pacing is associated with lower levels of disability (Nielson and Jensen, 2004), but other studies found Pacing is associated with higher levels of disability (McCracken and Samuel, 2007; Karsdorp and Vlaeyen, 2009; Kindermans, 2011). In a previous pilot study in which activity pacing behaviors and symptoms of pain and fatigue were measured using ecological momentary assessment (Murphy et al., 2008a), we found that people used pacing more frequently as symptoms were increasing throughout a day instead of using pacing as a pre-planned strategy as would be taught in chronic pain management programs. From these findings, we surmise that the natural use of pacing may be associated with higher levels of disability that may reflect the need to cope with problematic symptoms.

### Coping strategies and their relationship to activity patterns

An understanding of coping strategies and how they contribute to disability over time can influence the design of effective treatments and help to understand how best to avoid pathways to disability. Based on the theoretical models presented, physical activity levels are expected to be lower for avoiders compared to non-avoiders (Vlaeyen and Linton, 2000; Hasenbring and Verbunt, 2010). People who have higher use of task persistence may have higher activity levels compared to those who have lower use of task persistence (Hasenbring and Verbunt, 2010), although in some cases this task persistence could reflect overactivity. People who use pacing may have lower activity levels overall and this relationship was demonstrated in one pilot study (Murphy et al., 2008a). In addition, it is suggested that people who pace less frequently or who excessively persist in activities may have activity patterns that are more variable due to having to recover from overactive periods (Birkholtz et al., 2004; Huijnen et al., 2009).

Current research supports the notion that specific coping strategies can be associated with greater or diminished physical activity levels and activity patterns (Hasenbring et al., 2006; McCracken and Samuel, 2007; Murphy et al., 2008a; Huijnen et al., 2011). For example, people with chronic pain classified as avoiders or as pacers had lower levels of self-reported “up-time” (the hours spent standing or walking daily; McCracken and Samuel, 2007), and task persisters had higher levels of up-time when measured subjectively or objectively (McCracken and Samuel, 2007; Huijnen et al., 2011). In another study, people with low back pain 6 months after disk surgery were classified into subgroups of copers (fear-avoidant, endurance, or adaptive), and their activity was sampled over a day using a triaxial accelerometer. They found that endurance copers (those thought to be at risk for excessively persisting in activities) did not have a significantly different activity levels than adaptive copers, but they had a higher numbers of static strain postures during the day (such as sitting or standing with or without forward bending; Hasenbring et al., 2006). These findings suggest that in addition to activity levels, fluctuations or variability in activity patterns may provide important information about how people engage in activity as they cope with their symptoms. Huijnen et al. (2009) measured within-day activity fluctuations by having people with chronic low back pain categorize their activity into activity types reflecting different levels of effort (e.g., exercise vigorously – sitting or lying down) and found that increased within-day activity fluctuations were associated with disability whereas mean activity level was not. The relationship between coping and physical activity appears complex and is further complicated by the heterogeneous samples of people with different chronic pain conditions and the use of several different coping scales as well as different physical activity assessment methods. This study addressed a clear gap in this literature by examining how coping, symptoms, and physical activity are associated in people with OA.

To develop behavioral treatments for people with OA, our group has been investigating how momentary (i.e., within-day) symptoms relate to physical activity patterns and have found that while pain is related to activity, fatigue is more severe, more variable, and more negatively related to objective physical activity compared to pain (Murphy et al., 2008b). In order to examine how coping is associated with symptoms and activity, we examined both pain-activity and fatigue-activity relationships in this study in a sample of adults with knee or hip OA. Coping strategies were assessed with the Chronic Pain Coping Inventory (CPCI; Jensen et al., 1995) representing the areas of avoidance (Guarding, Resting, Asking for Assistance), Task Persistence, and Activity Pacing. According to the existing literature, we first hypothesized that Task Persistence would be associated with higher average levels of physical activity, whereas coping strategies that reflect avoidance and Pacing would be associated with lower average levels of physical activity. Second, we hypothesized that pacing would be associated with more stable, less variable levels of activity. Third, we hypothesized that coping strategies would moderate the association between pain/fatigue and activity. Specifically, we expected that people with high levels of Task Persistence would display weaker associations between pain/fatigue and activity compared to people with low levels of Task Persistence.

## Materials and Methods

This is a secondary data analysis of data from a larger three-arm randomized controlled trial (Murphy et al., 2011). The overall goal of the trial was to examine the effectiveness of a tailored activity pacing intervention delivered by occupational therapists for adults with symptomatic knee or hip OA. In the trial, participants were randomized into the tailored activity pacing intervention, general activity pacing, or usual care. Assessments occurred at baseline, posttest, and 6 months. Data from the baseline assessment period were used for these analyses. Ethical approval for this study was obtained by the University of Michigan Medical School Institutional Review Board and the Subcommittee on Human Studies in the Veteran’s Affairs Ann Arbor Healthcare System.

### Participants and procedures

Community-living veteran and non-veteran participants in this study sample were recruited from the University of Michigan and VA clinics, senior housing sites, and through public advertisements. Inclusion and exclusion criteria were designed to identify a cohort of community-living adults who were experiencing symptoms specifically due to their OA. Participants were included if they were age 50 years or older, had pain for at least 3 months duration, reported mild to moderate pain on the Western Ontario McMaster Arthritis Index (WOMAC) pain scale (Bellamy et al., 1988; Goggins et al., 2005), had radiographic evidence of knee or hip OA (Kellgren–Lawrence scale of ≥2; Kellgren and Lawrence, 1957), had adequate cognitive status (evidenced by scoring ≥5 on the six item cognitive screener (Callahan et al., 2002), could reliably operate the Actiwatch-S accelerometer used in the study, and were English-speaking. Participants were excluded if they: had medical conditions that could interfere with pain and fatigue reporting or activity monitoring (e.g., multiple sclerosis, lupus, rheumatoid arthritis, Parkinson’s disease); were diagnosed with cancer in the last year (other than skin cancer) or were currently undergoing treatment for cancer; had medicine changes within the previous 2 weeks, anemia or unmanaged thyroid dysfunction (from blood work result); had two or more days of complete bed rest within last month; had limb hemiplegia or amputation; underwent a knee arthroscopic procedure within the previous 2 months; underwent replacement of any hip or knee within the last 6 months; knee joint injections within the previous 3 months; current receipt of physical or occupational therapy for OA symptoms or knee/hip problems; or currently or recently attended (in the previous 12 months) a cognitive behavioral program or other self-management program that included activity pacing instruction.

To determine eligibility, study personnel first screened potential participants by phone. If eligible based on screening, potential participants were scheduled for a clinic visit to undergo an x-ray of their knees or hips, complete questionnaires, learn how to operate the Actiwatch-S accelerometer, and complete physical performance testing. Participants signed a written consent form at the clinic visit and after completion of that visit, eligible participants were mailed the Actiwatch-S accelerometer and corresponding logbook to undergo the baseline 7 day home monitoring period. For the home monitoring period, participants wore the Actiwatch-S on their non-dominant wrist. The Actiwatch-S collects physical activity data and allows for time-stamped participant-entered responses. Participants were instructed on how to enter responses into the accelerometer using a standardized interactive learning module and were given the opportunity to practice rating their symptoms using the accelerometer’s input button to record the information. Participants also became familiar with the logbook that accompanied the accelerometer. The logbook was used to cross-validate the items and served as a back-up if there were missing data from the accelerometer.

A total of 47 participants were enrolled in the study and data from 44 were used in these analyses. One individual was eliminated due to completely missing momentary data (activity, pain, fatigue). One individual was identified as an outlier based on body mass index (BMI; in which BMI = 55.99, nearly 16 points higher than the next highest individual). Another individual who was identified as an outlier was experiencing a “pain flare” with pain ratings of 8/10 compared to “typical” 4–5/10 pain intensity during the study and was consequently unable to finish the physical performance testing [e.g., Timed Up and Go (TUG)].

### Measures

#### Primary measures

Coping strategies were measured at the baseline clinic visit using the CPCI, a self-report measure of cognitive and behavioral strategies for coping with pain. The CPCI has demonstrated excellent test-retest reliability and internal consistency and concurrent validity in a chronic pain sample (Jensen et al., 1995). The original questionnaire consisted of 65 items divided into eight subscales: four of which were originally considered wellness-based coping (Task Persistence, Exercise, Relaxation, and Coping Self-Statements), three were considered illness-based coping (Guarding, Resting, Asking for Assistance), and one was neither (Seeking Social Support). A later version included activity pacing as a subscale (Nielson et al., 2001) which was included in the version used in this study. The scores for each item range from 0 to 7 days, with a score of 0 indicating that the participant did not use that coping strategy in the past week. For this analysis, we chose only the subscales that could be best equated to activity patterns identified in the cognitive behavioral models: avoidance behaviors (Guarding, Resting, Activity for Assistance), Task Persistence, and Pacing. These subscales all had adequate internal consistency in the present sample (Cronbach’s alpha ranging from 0.72 to 0.82).

Physical activity patterns were measured using a wrist-worn accelerometer, the Actiwatch-S [Philips Respironics, Mini-Mitter, Bend, OR, USA] that measures changes in acceleration. Although it is worn on the wrist, it is highly associated with whole-body movement (Patterson et al., 1993; Westerterp, 1999). The Actiwatch-S has been shown to have excellent inter-unit reliability (*r* = 0.98) and preliminary criterion validity among a sample of chronic pain patients (Gironda et al., 2007). The device records changes in acceleration every 15 s and these are recorded as activity counts. Higher activity counts generally reflect participation in higher intensity activities (Swartz et al., 2000; Murphy, 2009). Physical activity data from the accelerometer are aggregated in different ways. Daytime activity counts were aggregated within a day between symptom reporting periods (which we call “momentary activity”). These are roughly 4 h intervals between wake-up and 11:00 am; 11:00 am to 03:00 pm, 03:00 pm to 07:00 pm, and 07:00 pm to bedtime). Activity counts were also aggregated for each day (i.e., daily activity) and over the 7 day period for each person. Because participants wear the accelerometer continuously for 7 days, it was necessary to establish participants’ wake-up and bed times. A previously established algorithm was used to corroborate participant report with the objective measures (Murphy et al., 2008b).

Momentary pain and fatigue severity were measured on 0–10 numerical rating scales that were directly input into the accelerometer five times a day [at rise time in the morning, three times during waking hours (11:00 am, 03:00 pm, 07:00 pm), and at bedtime]. The accelerometer was worn for 7 days at baseline and at the outcome assessment periods. Pain was rated on a scale from 0 = no pain to 10 = pain as bad as you can imagine. Fatigue, defined as tiredness or weariness (Wolfe et al., 1996), was rated on a scale from 0 = no fatigue to 10 = fatigue as bad as you can imagine.

#### Background measures and covariates

Background demographics included age, sex, marital status, ethnicity, race, employment status, veteran status, and educational level. Health status variables included BMI and OA disease severity as determined from a radiologist’s scaling on the Kellgren–Lawrence Scale using the joint x-rays (Kellgren and Lawrence, 1957).

To assess general symptoms and reported physical and mental health, several scales were used. To measure symptom severity and interference, the Brief Pain Inventory (BPI; Keller et al., 2004) and Brief Fatigue Inventory (BFI; Mendoza et al., 1999) were used. Depressive symptoms were measured using the Center of Epidemiologic Studies Depression Scale (CES-D; Radloff, 1977). The State Trait Personality Inventory (STPI) was used to measure anxiety symptoms (Spielberger et al., 1970). The Short Form 12 was also used to measure mental and physical health domains (Ware et al., 1996). The WOMAC physical disability subscale was also used as a measure of arthritis-related physical function (Bellamy et al., 1988). Internal consistency on all of these scales was acceptable ranging from 0.74 on the CES-D to 0.92 on the BFI.

Physical function was measured using two validated objective measures. The TUG test (Podsiadlo and Richardson, 1991) measures the time (in seconds) to get up from a chair, walk 20 feet, and return to the chair. Participants completed three trials and the average was used in the analyses. The 6 min walk test is a walking test in which people are asked to walk at their usual pace for 6 min and overall distance was recorded in feet (Butland et al., 1982).

#### Data analysis

Descriptive statistics for all predictor and outcome variables were calculated and examined for distribution normality. Skew (largest range was for TUG = 0.06–1.64) and kurtosis (TUG range = 0.12–4.13) values indicated that all variables were sufficiently normally distributed to conduct the primary analyses (West et al., 1995). To address these modest deviations from normal distribution in the primary analyses, we utilized an asymptotically consistent estimator, the “sandwich estimator,” which counteracts problems due to non-normality in the data by generating robust standard errors analyses (described below; Huber, 1967; White, 1980).

Multilevel random effects modeling (MLM) was used to test the study hypotheses. This statistical approach is optimal because these data have a hierarchical structure with momentary evaluations (Level 1) nested within days (Level 2) nested within individuals (Level 3). MLM, using the SAS PROC MIXED procedure can simultaneously model between- (Level 3) and within-person (Levels 1 and 2) variance, can account for auto-correlation between adjacent observations, and has contemporary techniques for addressing missing data (e.g., all available data points are used, cases are not eliminated due to missing Level 1 or 2 data). In addition, with MLM we were able to model random effects, which assume that the independent variable represents a random sample of a larger range of possible values, in addition to fixed effects, which assume that all possible values are represented in the independent variable. Modeling of random effects allows for generalization of results to a broader population of people compared to simple fixed effect analyses. Prior to conducting MLM analyses, variables were centered, based on guidelines for centering data in multilevel statistical procedures (Enders and Tofighi, 2007). Momentary variables were person-day-centered such that values indicate change from a person’s average for that day and between-person variables were sample-centered such that values indicate difference from the sample-average. All analyses were conducted using SAS statistical software, Version 9.2 (SAS Institute Inc. 2009. SAS OnlineDoc^®^ 9.2. Cary, NC, USA: SAS Institute Inc.).

To test the first set of questions, the association between coping variables and activity level (H1) and activity variability (H2), two separate multilevel models were constructed. For the first model, all coping variables (Guarding, Resting, Asking for Assistance, Task Persistence, Pacing) were included as predictors simultaneously to predict average momentary activity. For the second model, average activity values were aggregated at the level of a day and the standard deviation of activity for each day was constructed as an indicator of activity variability. *Post hoc* analyses to determine whether the association between coping and activity level and activity variability differed by average level of activity (e.g., highly active versus sedentary) were conducted by testing interaction terms [(COPING) × MEAN ACTIVITY] in the prediction of activity and activity variability. In all models, including the ones described below, age, BMI, TUG score, and KL score were included as covariates.

To test the second set of hypotheses, regarding the moderating effect of coping on the pain/activity and fatigue/activity (H3) associations, MLM models were constructed with interaction terms [e.g., (COPING) × PAIN/FATIGUE] entered as predictors of momentary average activity. Significant interactions were further examined through graphing the simple slopes between pain/fatigue and activity for low (−1 SD), mean, and high (+1 SD) values of the moderating variables (i.e., coping; Aiken and West, 1991).

## Results

Complete sample descriptive statistics are depicted in Table 1. Results indicate that the sample reported “mild” levels of pain intensity, fatigue, and stiffness and was, on average, obese according to BMI scores. The sample was mostly white (81.8%), female (68%), non-veteran (79.5%), and married (59.5%). For physical function, the sample had an average of 9.1 s on the TUG test which is slightly slower than normative values of people aged 60–69 (Bohannon, 2006), but comparable to a previous sample of women with knee or hip OA (Murphy et al., 2008b). The 6 min walk distance (1249 feet or 381 m) was also slightly slower than norms for healthy adults that range from 400 to 700 m (Enright, 2003).

**Table 1:** **Sample demographics (*N* = 44)**.

Variable	Mean	SD	Min	Max
Age	66.48	6.93	53	84
BMI^†^	30.81	5.01	23.34	43.36
TUG^‡^	9.10	1.86	5.51	16.81
6 min walk	1248.82	224.73	825	1930
Pain (0–10)*	2.98	1.45	0.61	6.46
Fatigue (0–10)*	3.26	3.03	0.71	7.34
Stiffness (0–10)*	3.42	1.52	0.82	7.23
Activity	348.34	83.33	200.88	541.97
			*N*	%
Sex	Male		14	31.8
	Female		30	68.2
Marital status	Single never married		1	2.4
	Married		25	59.5
	Divorced		9	21.4
	Widowed		7	16.7
Ethnicity	Non-Hispanic		43	100
Race	American Indian/Alaskan native		1	2.3
	Black/African American		5	11.4
	White		36	81.8
	More than one race		2	4.5
Employment status	Working/volunteering ≥ 36 h/week		6	13.6
	Working/volunteering 20–35 h/week		7	15.9
	Retired, not working at least 20 h/week		25	56.8
	Other		6	13.6
Veteran status	Non-veteran		35	79.5
	Veteran		9	20.5
Educational level	12 years		5	11.9
	13–16 years		16	38.1
	17–20 years		18	42.9
	21–25 years		3	7.2

*Variable represents average across the ecological momentary assessment period; ^†^one individual with BMI = 55.99 was identified as an outlier and removed from main analyses; ^‡^one individual was unable to complete the TUG in the maximum allotted time of 30 s and these data were not included in analyses.

Prior to conducting the analyses to address the primary study aims, we examined the distribution and correlation of the CPCI coping subscales with each other and with key indicators of functioning. As can be seen in Table 2, the CPCI subscales were not generally highly correlated with each other. Some notable exceptions were significant positive correlations between Activity Pacing and Guarding and Resting, a significant positive correlation between Asking for Assistance and Guarding, and a significant negative correlation between Task Persistence and Guarding. Task Persistence was the most commonly reported coping strategy, averaging nearly 4.5 days/week in this sample. The least commonly used coping strategy was Asking for Assistance, which averaged less than 2 days/week in this sample. Correlations with measures of pain and mental and physical health (Table 3) indicate a few significant correlations with coping subscales. Guarding showed a positive and moderate association with pain and fatigue intensity/impact and with physical dysfunction. Resting was positively associated with both fatigue and physical dysfunction measures. Asking for Assistance was similarly positively correlated with measure of pain and fatigue and was also associated with greater depressive symptoms. Task Persistence was negatively correlated with depressive symptoms. Pacing was the only activity scale to correlate with less anxiety and greater mental health, but was also related to greater physical dysfunction.

**Table 2:** **Correlations and distribution statistics for CPCI subscales (*N* = 44)**.

CPCI subscales	Guarding	Resting	Asking for assistance	Task persistence	Pacing
Guarding	–				
Resting	0.28	–			
Asking for assistance	0.40**	0.14	–		
Task persistence	−0.31*	0.01	−0.17	–	
Activity pacing	0.33*	0.30*	0.07	0.08	–
Mean	3.40	3.18	1.63	4.47	3.68
SD	1.38	1.45	1.60	1.34	1.67
Skew	−0.18	0.06	0.63	−0.17	0.33
Kurtosis	−0.28	−0.17	−0.86	−1.12	−0.29

*p ≤ 0.05, **p < 0.01.

**Table 3:** **Correlations between CPCI subscales and measures of pain, mental, and physical health**.

	Guarding	Resting	Asking for Assistance	Task persistence	Activity pacing
**PAIN**					
BPI-total score	0.49**	0.29	0.45**	−0.26	0.23
**FATIGUE**					
BFI-total score	0.41**	0.47**	0.44**	−0.28	−0.04
**MENTAL HEALTH**					
CES-D (depressive symptoms)	0.29	0.17	0.36*	−0.38**	−0.27
STPI (anxiety)	−0.19	0.10	0.15	0.08	−0.33*
SF-12 mental component score	0.05	0.12	0.03	0.09	0.32*
**PHYSICAL HEALTH**					
WOMAC (physical dysfunction)	0.35*	0.35*	0.29	−0.21	0.32*
SF-12 physical component score	0.01	0.12	0.04	0.09	0.28

*p ≤ 0.05, **p < 0.01. BPI, Brief Pain Inventory; BFI, Brief Fatigue Inventory; CES-D, Center for Epidemiological Studies Depression; STPI, State Trait Personality Inventory; SF-12, Short Form-12; WOMAC, Western Ontario McMaster Arthritis Index.

### Coping and activity level and variability

Results for the MLM predicting objective physical activity from momentary pain and fatigue, coping subscales, controlling for demographic and clinical variables indicated that among the covariates, only BMI was significantly related to lower activity levels (*p* = 0.01). Momentary pain was not related to activity level, but momentary fatigue was negatively related to activity (*p* < 0.001). In terms of coping, Guarding was significantly related to lower activity levels (*p* = 0.03) and Asking for Assistance was significantly related to higher activity levels (*p* < 0.001). No other coping subscales approached significance in predicting activity level. We also examined whether average activity level (i.e., whether someone was generally inactive or active) moderated the association between coping subscales and activity level by including interaction terms AVERAGE ACTIVITY × (COPING) in the equation. In no case did average activity level moderate the association between coping and momentary activity. All of these results were replicated when we ran the MLM with all coping subscales included and separate MLMs for each subscale (to optimize power to detect differences, given our small *n*).

Results for the MLM predicting activity variability indicated that no demographic or clinical variables were significant predictors. In direct contrast to the findings for activity level, pain (est. = −1.52, SE = 0.63, *t* = −2.40, *p* = 0.02) but not fatigue predicted activity variability – lower pain was associated with higher activity variability. No coping variables were significant predictors of between-person variability in activity. As with the analyses for activity level, we also examined whether average activity level moderated the association between coping and activity variability. In no case did average level of activity moderate the association between coping and activity variability. All of these findings were consistent whether we ran the MLM with all coping subscales included or where we ran separate MLMs for each subscale (to optimize power to detect differences, given our small *n*).

### Coping as a moderator of the association between pain and activity

**Figure 1:**
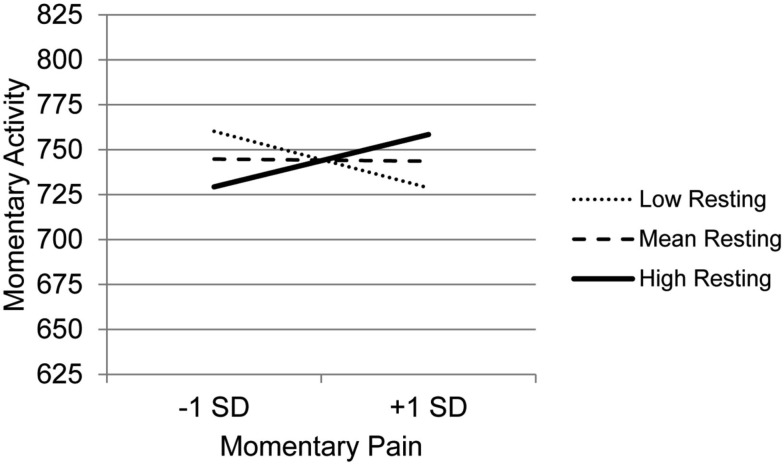
**Simple regression slopes for centered momentary pain and use of Resting at low, mean, and high levels of momentary physical activity**.

Results for the MLM testing whether the various types of coping moderate the association between momentary changes in pain and activity (Table 4) indicate that BMI is the only clinical variable that was a significant predictor, showing a negative association with activity. In terms of main effects of coping, consistent with the findings for the first research question, Guarding was significantly related to less activity and Asking for Assistance was related to more activity. Only one coping variable, Resting, moderated the association between pain and activity. Examination of the simple slope between momentary changes in pain and momentary activity at low, mean, and high use of Resting (Figure 1), indicate that for those who more frequently use Resting as a coping strategy, there is a positive association between momentary changes in pain and activity. For those with mean levels of use of Resting, there is little association between pain and activity, whereas for those who use Resting infrequently, there is a negative association between pain and activity. In other words, those who experience increases in pain with increased activity, are more likely to use resting as a means of coping that those who either do not experience a relation between pain and activity or who experience increases in pain in the context of lower activity (e.g., such as during resting). However, it is important to note that we cannot determine from these data whether resting causes a stronger association between pain and activity or whether the strong association between pain and activity precipitates resting behavior.

**Table 4:** **Multilevel model results predicting momentary activity from interaction terms including coping subscales and pain**.

Covariance parameter	Subject	Estimate	SE	*Z*	*p*
**RANDOM EFFECTS**					
Intercept UN	ID	6720.36	2263.41	2.97	<0.01
AR(1)	ID	0.09	0.04	2.50	0.01
Residual		19792	887.11	22.31	<0.0001

**Effect**		**β**	**SE**	***T***	***p***

**FIXED EFFECTS**					
Intercept		744.21	130.44	5.71	<0.0001
Level 1 (df = 30)	
Age		−2.67	1.67	−1.60	0.12
BMI		−6.85	2.28	−3.00	0.01
		3.20	9.39	0.34	0.74
KL		−26.29	26.44	−0.99	0.32
Average pain		3.22	9.19	0.35	0.73
Guarding*		−30.07	13.06	−2.30	0.03
Resting*		−0.21	8.02	−0.03	0.98
Asking for assistance*		39.09	10.42	3.75	<0.001
Task persistence*		11.54	14.78	0.78	0.44
Activity pacing*		−1.00	10.65	−0.09	0.93
Level 3 (df = 1032)	
ΔPain		−0.50	4.80	−0.10	0.92
Level 1 × Level 3 (df = 1032)	
ΔPain × guarding		5.69	3.30	1.73	0.09
ΔPain × resting		8.99	4.55	1.98	0.04
ΔPain × asking for assistance		−4.43	3.73	−1.19	0.24
ΔPain × task persistence		1.21	4.03	0.30	0.76
ΔPain × activity pacing		−1.46	2.91	−0.50	0.62

*Scales from the Chronic Pain Coping Inventory.

### Coping as a moderator of the association between fatigue and activity

**Figure 2:**
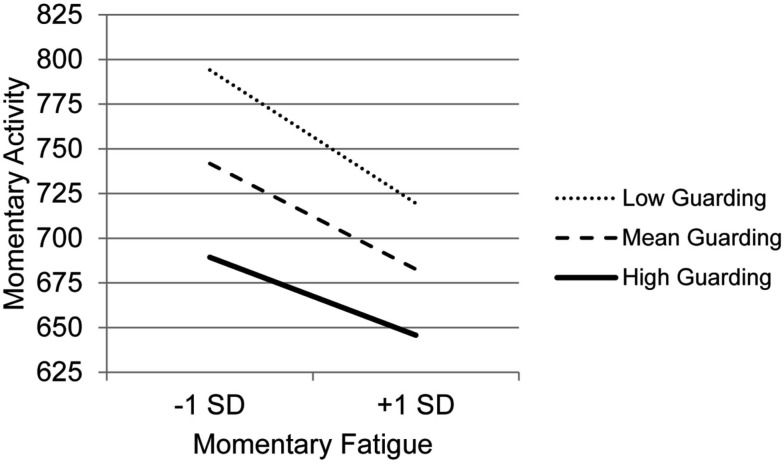
**Simple regression slopes for centered momentary fatigue and use of Guarding at low, mean, and high levels of momentary physical activity**.

Results for the MLM testing whether the various types of coping moderate the association between momentary changes in fatigue and activity level (Table 5) indicate that BMI is the only clinical variable that was a significant predictor – those with higher BMI had lower levels of activity. In terms of main effects of coping, consistent with the findings for the first study question, Guarding was significantly related to less activity and Asking for Assistance was related to more activity. In contrast to the findings for pain, four coping subscales moderated the association between momentary changes in fatigue and activity: Guarding, Resting, Task Persistence, and Pacing. The graph of the moderating effect of Guarding (Figure 2), which also clearly shows that higher levels of guarding are related to lower levels of activity, indicates that with decreasing use of Guarding, the association between momentary changes in fatigue and activity is more negative. Those who use Guarding the most have the lowest level of activity and the lowest association between momentary fatigue and activity.

**Table 5:** **Multilevel model results predicting momentary activity from interaction terms including coping subscales and fatigue**.

Covariance parameter	Subject	Estimate	SE	*Z*	*p*
**RANDOM EFFECTS**					
Intercept UN	ID	6991.39	2400.42	2.91	0.002
AR(1)	ID	0.07	0.04	1.95	0.05
Residual		18649	834.02	22.36	<0.0001

**Effect**		**β**	**SE**	***T***	***p***

**FIXED EFFECTS**					
Intercept		712.15	137.59	5.18	<0.0001
Level 1 (df = 30)	
Age		−2.22	1.78	−1.25	0.22
BMI		−6.67	2.33	−2.86	0.01
		4.09	9.68	0.42	0.68
KL		−29.38	28.42	−1.03	0.30
Average fatigue		1.31	6.34	0.21	0.84
Guarding*		−31.22	13.35	−2.34	0.03
Resting*		−0.60	8.32	−0.07	0.94
Asking for assistance*		40.09	11.09	3.62	0.001
Task persistence*		13.43	15.05	0.89	0.38
Activity pacing*		−1.28	10.02	−0.13	0.90
Level 3 (df = 1033)	
ΔFatigue		−20.52	2.95	−6.96	<0.0001
Level 1 × Level 3 (df = 1033)	
ΔFatigue × guarding		3.75	1.72	2.18	0.03
ΔFatigue × resting		4.89	1.73	2.82	0.005
ΔFatigue × asking for assistance		−3.56	2.06	−1.72	0.09
ΔFatigue × task persistence		6.41	1.99	3.22	0.001
ΔFatigue × activity pacing		3.84	1.16	3.31	0.001

*Scales from the Chronic Pain Coping Inventory.

**Figure 3:**
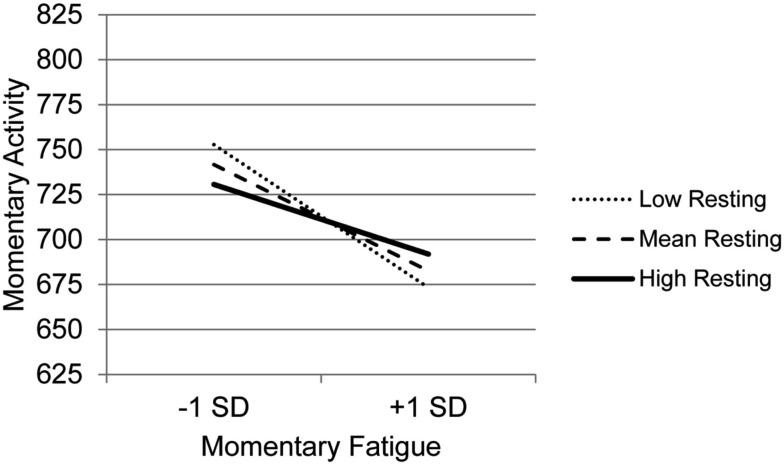
**Simple regression slopes for centered momentary fatigue and use of Resting at low, mean, and high levels of momentary physical activity**.

**Figure 4:**
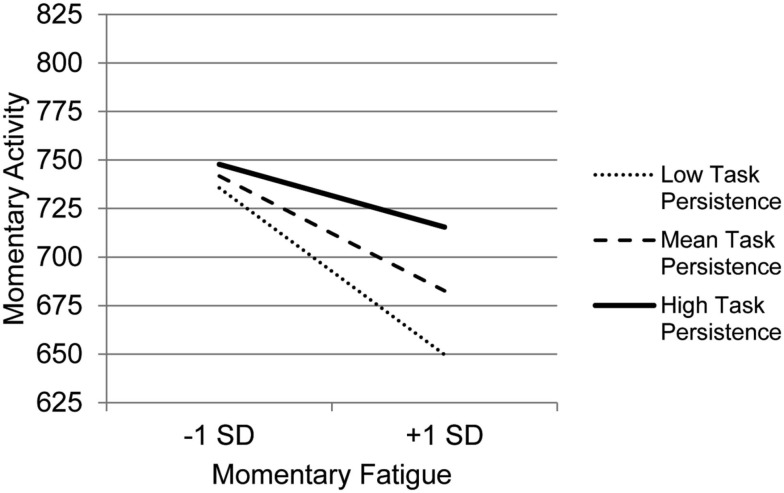
**Simple regression slopes for centered momentary fatigue and use of  Task Persistence at low, mean, and high levels of momentary physical activity**.

**Figure 5:**
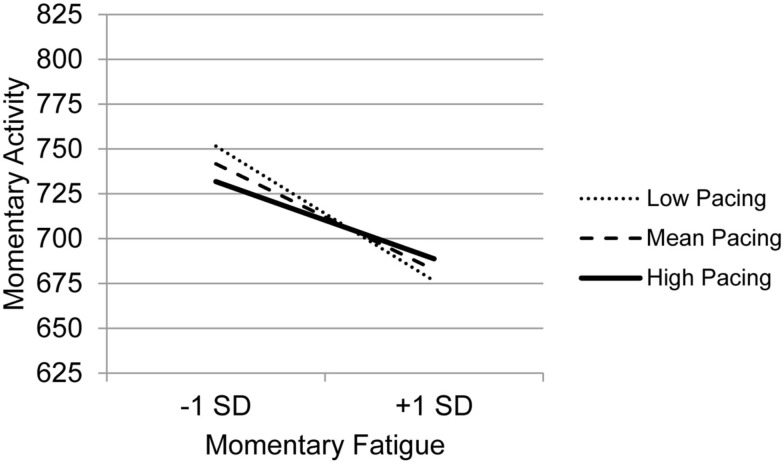
**Simple regression slopes for centered momentary fatigue and use of Pacing at low, mean, and high levels of momentary physical activity**.

The graph for Resting (Figure 3) indicates that with decreasing use of Resting, the association between fatigue and activity is increasingly negative. Those who experience the most precipitous drops in activity in the context of high fatigue are the least likely to use Resting as a coping behavior, whereas those who report frequent resting do not show as steep a decline in activity in the context of increased fatigue. The graph for Task persistence (Figure 4) indicates that those who with the highest use of Task Persistence have the lowest association between momentary fatigue and activity; the fatigue/activity association becomes increasingly negative with decreasing use of Task Persistence. In other words, those who report high levels of persistence have slightly lower activity levels in the context of increased fatigue; in contrast, those who report low persistence show a rather steep drop in activity with increased fatigue. The graph for Pacing (Figure 5) indicates a similar, though less dramatic pattern. Those with the highest use of Pacing have the smallest association between momentary fatigue and activity. With decreasing use of Pacing, the association between fatigue and activity becomes increasingly negative.

## Discussion

In this study, we investigated the relationship between symptoms of pain and fatigue, coping strategies, and objective activity patterns over a 7 day period in a sample of adults with knee or hip OA. There is a paucity of research that has examined how coping relates to the association between symptoms and activity in OA, and the use of ecological momentary assessment in this study provides important insights and brings up additional research questions.

We first examined the relationships between symptoms, coping, and activity in separate models for each symptom (pain and fatigue). We found that pain assessed momentarily was not significantly related to activity levels as measured by accelerometer. This finding is similar to previous studies of low back pain that have also reported a lack of relationship between pain and activity when measured objectively (Vendrig and Lousberg, 1997; Hasenbring et al., 2006; Huijnen et al., 2010). Fatigue, a symptom that is not typically addressed in clinical interventions and not typically examined in OA research studies, was significantly and negatively related to activity levels similar to our findings in a separate sample of women with knee or hip OA (Murphy et al., 2008b). In addition, higher BMI was associated with lower activity levels in all statistical models. Given that many people with knee or hip OA often have high BMI and physical activity interventions are widely recommended for this population, it may be important to address fatigue management in these interventions given its strong association with lowered activity levels.

In these models, only two coping strategies that we initially classified as avoidance behaviors, Asking for Assistance and Guarding, were significantly associated with activity levels, but in opposite directions. Consistent with our hypothesis, Guarding was associated with lower activity. In contrast with expectations; however, Asking for Assistance was associated with higher activity levels. People with the lowest activity levels had the highest use of Guarding and the lowest use of Asking for Assistance. These findings are interesting given that both of these coping strategies were highly associated with pain interference on the BPI. It may be that people who are asking for assistance have more opportunities to interact and ask others for help; however, Asking for Assistance was also associated with depressive symptoms on the CES-D. While Asking for Assistance appears to be associated with a number of negative outcomes, the findings of this study suggest that it is not associated with low levels of activity as one might expect. Since this was the least commonly used strategy from a relatively small sample, future work should further examine how this behavior impacts symptoms and functioning.

When we examined the relationships between symptoms, coping, and activity variability, we found that only pain was negatively associated with activity variability, suggesting that those who have relatively high levels of pain maintain a more consistent level of activity across a day than those who experience less pain. No coping variables were associated with activity variability. Some studies suggest that increased activity variability is associated with poor outcomes (Huijnen et al., 2009; Kindermans et al., 2011). As a result, activity pacing is often encouraged as one solution to high variability in daily activity. People who use pacing (Fordyce, 1976) should have less variability in their activity as they are trying to maintain a steady pace and reduce periods of high activity that could lead to a flare in symptoms (Birkholtz et al., 2004). In a pilot study, we found that people who participated in a tailored activity pacing intervention had reduced variability in their activity and reduced fatigue levels after the intervention while maintaining similar average activity levels found at baseline (Murphy et al., 2012). However, it is important to note that pacing could potentially be viewed as an adaptive or maladaptive behavior depending upon whether or not people are instructed how to pace (Murphy and Clauw, 2010). For instance, some studies have found positive associations between the natural use of pacing and measures of disability (McCracken and Samuel, 2007; Karsdorp and Vlaeyen, 2009; Kindermans et al., 2011). Therefore, it is notable that self-reported natural levels of pacing are not related significantly to daily variability in activity. More research is needed to determine how the use of pacing (both use of pacing naturally and after pacing instruction) affects physical activity variability over longer periods of time.

### Coping as a moderator variable

Tests of interaction effects in this study are important because they examine some key assumptions about how the behavioral coping strategies under consideration work on a moment to moment basis. For example, it is thought that when people experience days or moments of high pain or high fatigue, these symptoms will affect their level of activity. The assumptions underlying the avoidance, persistence, pacing categories is that use of these coping strategies will modify the expected relationship between pain or fatigue and activity. Avoidance strategies are thought to encompass a group of behaviors that result in restricted activity (rest, guarding, asking for assistance) in the context of symptoms. Persistence strategies are thought to reflect independence of activity and symptom severity due to the assumption that a person who persists carries on despite their discomfort. Pacing, like persistence, is thought to reflect a strategy of intentionally planning and carrying out activities, somewhat independent of pain or fatigue severity. This is the first known test of these assumptions about the types of coping. We found that our hypotheses were partially supported and that the effects of the moderators were different depending on whether we were examining pain or fatigue.

For the relationship between pain and activity, only Resting was a significant moderator. People who use Resting most frequently had a positive relationship between pain and activity. It appears that people who rest the most frequently have the highest levels of activity-related pain, whereas people who rest the least have a negative relationship between activity and pain. It seems sensible to conclude that those who experience that greatest increases in pain with increased activity might be more prone to rest as a means of attempting to cope with the pain. It is possible that people who use Resting the least may get relief from pain with activity and/or slight increases in pain with lower activity (e.g., resting), either due to physiological processes or psychological processes such as being distracted from pain by high activity, but further study is needed to examine the nature of this relationship. Though this seems like the most plausible explanation, given the correlational nature of our data and analyses and the fact that the association between pain and activity is the opposite of what we had expected, we cannot draw causal conclusions from these data. Further examination that looks at moment to moment dynamic associations between resting as a means of coping, changes in pain, and activity level would help to delineate the direction of these associations.

For the relationship between fatigue and activity, several coping variables were significant moderators. Although we expected that people who most frequently use coping strategies considered avoidant would have the strongest relationships between symptoms and activity, high levels of Guarding and resting were associated with the weakest associations between fatigue and activity. Specifically people who use Guarding most frequently had the smallest association between fatigue and activity. However, because we found that people who use Guarding are less active than people who use other coping strategies, it may be that these people do not engage in activity at a level that increases fatigue. Taken together with the findings for the moderating effect of resting on the pain/activity association, these findings suggest that use of avoidant coping strategies may be driven by increases in pain or fatigue in the context of activity. This is in contrast to the notion that people select a coping strategy (based on training or natural inclination) and that coping strategy is a main determinant of the experience of symptoms and activity. Though these data are considered preliminary and in need of further examination, these findings may suggest that the direction of the relationship between some coping strategies and symptoms/activity that we are observing here are different from what is commonly assumed.

Turning to persistence coping, we hypothesized that people who most frequently use task persistence would have the lowest relationship between fatigue and activity level and this was supported. People who reported high levels of persistence had the highest activity levels and for these people there was a dissociation between symptoms and activity such that engaging in activity was not dependent on having low fatigue. For people who used Task Persistence less, when fatigue was high, there was a steep decline in activity level. This finding is significant in that it provides objective evidence that people who say they persist in tasks are actually doing so in terms of persisting through their fatigue, and seems to support other studies in which Task Persistence as measured by the CPCI is considered a positive coping strategy (Jensen, 1991;Jensen et al., 1995). It is important to note that of all the CPCI subscales that we examined, only Task Persistence showed negative correlations with pain and fatigue. Although these correlations were small and non-significant in this sample, these data may suggest that Task Persistence is more feasible for those with relatively lower pain or that Task Persistence somehow results in lower pain intensity and fatigue. Also notable is that Task Persistence was moderately negatively related to depressive symptoms. As with the other symptoms, it is both possible that those who are not depressed find it easier to persist than those with greater depression or, alternatively, that persisting through tasks results in better mood.

The use of Pacing also moderated the relationship between fatigue and activity. While all groups of pacers had negative relationships between fatigue and activity, the people who most frequently used activity pacing had the weakest relationship between fatigue and activity. Whether a person used pacing frequently or not, increases in fatigue were related to decreases in activity; but, for those with the highest level of pacing behaviors, the drop in activity was less severe compared to those who reported low levels of pacing. Like Task Persistence, Pacing was related to indicators of positive mental health. Specifically it was related to lower levels of anxiety and higher overall mental health. Given that we cannot infer causation from these data, it is possible that pacing is more feasible strategy for those who have better mental health, or that something about Pacing behavior promotes better mental health.

### Strengths and limitations

This is the first study to our knowledge that investigated how coping strategies relate to the association between symptoms and activity in people with knee and hip OA. As such, it is somewhat difficult to compare our findings to other studies. In addition, we are measuring activity patterns using a wrist-worn device which may yield different findings than studies that use devices worn on different sites of the body and that measure other variables such as position change or energy expenditure. The use of ecological momentary assessment of symptoms with concurrent physical activity reporting provides rich information on people’s daily life patterns. The use of the CPCI to measure coping strategies was an important starting point in this research; however, this measure which involves a 7 day recall of past coping strategies restricted our treatment of coping strategy as a personal trait. It is likely that people with OA have more complex coping strategy use, that is, they may not be only a “task persister” or an “avoider,” and selection of coping strategy may be depend on the particular situation. It should be noted that in this study, the CPCI, which asks participants to recall their coping during the previous 7 days, was completed at the baseline visit instead of the week of the home monitoring which could have potentially attenuated associations between coping strategy use, symptoms, and activity. Future studies should examine momentary use of coping strategies to better examine how within-day use of these strategies relates to subsequent physical activity and symptoms. Due to the fact that our sample was mostly white females diagnosed with OA, with low levels of pain and fatigue, our ability to generalize our findings to other populations is somewhat limited. Our conclusions regarding these findings would be strengthened by replication in other samples.

## Conclusion

In conclusion, we found that coping strategies moderated the relationship between pain and activity and fatigue and activity in different ways. Many coping strategies were moderators of how people engage in activity with fatigue. While most treatment efforts in OA are focused on pain, this study supports the importance of examining how people cope with fatigue in their daily lives to help develop treatments that also address this symptom.

## Conflict of Interest Statement

The authors declare that the research was conducted in the absence of any commercial or financial relationships that could be construed as a potential conflict of interest.

## References

[B1] Aiken L. West S. (1991). Multiple Regression: Testing and Interpreting Interactions. Thousand Oaks: Sage Publications, Inc

[B2] Bellamy N. Buchanan W. Goldsmith C. Campbell J. Stitt L. (1988). Validation study of WOMAC: a health status instrument for measuring clinically important patient relevant outcomes to antirheumatic drug therapy in patients with osteoarthritis of the hip or knee. J. Rheumatol. 15, 1833–1840 3068365

[B3] Birkholtz M. Aylwin L. Harman R. M. (2004). Activity pacing in chronic pain management: one aim, but which method? Part one: introduction and literature review. Br. J. Occup. Ther. 67, 447–452

[B4] Bohannon R. W. (2006). Reference values for the timed up and go test: a descriptive meta-analysis. J. Geriatr. Phys. Ther. 29, 64–68 10.1519/00139143-200604000-00001 16914068

[B5] Boutron I. Rannou F. Jardinaud-Lopez M. Meric G. Revel M. Poiraudeau S. (2008). Disability and quality of life of patients with knee or hip osteoarthritis in the primary care setting and factors associated with general practitioners’ indication for prosthetic replacement within 1 year. Osteoarthritis Cartilage. 16, 1024–1031 10.1016/j.joca.2008.01.001 18276169

[B6] Butland R. J. Pang J. Gross E. R. Woodcock A. A. Geddes D. M. (1982). Two-, six-, and 12-minute walking tests in respiratory disease. Br. Med. J. (Clin. Res. Ed.) 284, 1607–1608 10.1136/bmj.284.6329.1607 6805625 PMC1498516

[B7] Callahan C. Unverzagt F. Hui S. Perkins A. Hendrie H. (2002). Six-item screener to identify cognitive impairment among potential subjects for clinical research. Med. Care 40, 771–781 10.1097/00005650-200209000-00007 12218768

[B8] Enders C. Tofighi D. (2007). Centering predictor variables in cross-sectional multilevel models: a new look at an old issue. Psychol. Methods 12, 121–138 10.1037/1082-989X.12.2.121 17563168

[B9] Englbrecht M. Gossec L. Delongis A. Scholte-Voshaar M. Sokka T. Kvien T. K. Schett G. (2012). The impact of coping strategies on mental and physical well-being in patients with rheumatoid arthritis. Semin. Arthritis Rheum. 41, 545–555 10.1016/j.semarthrit.2011.07.009 22340997

[B10] Enright P. L. (2003). The six-minute walk test. Respir. Care 48, 783–785 12890299

[B11] Ersek M. (2006). Use of the chronic pain coping inventory to assess older adults’ pain coping strategies. J. Pain 7, 833–842 10.1016/j.jpain.2006.04.002 17074625

[B12] Fordyce W. (1976). Behavioral Methods for Chronic Pain and Illness. St. Louis: Mosby

[B13] Gironda R. J. Lloyd J. Clark M. E. Walker R. L. (2007). Preliminary evaluation of reliability and criterion validity of Actiwatch-Score. J. Rehabil. Res. Dev. 44, 223–230 10.1682/JRRD.2006.06.0058 17551874

[B14] Goggins J. Baker K. Felson D. (2005). What WOMAC pain score should make a patient eligible for a trial in knee osteoarthritis? J. Rheumatol. 32, 540–542 15742450

[B15] Grotle M. Hagen K. Natvig B. Dahl F. Kvien T. (2008). Prevalence and burden of osteoarthritis: results from a population survey in Norway. J. Rheumatol. 35, 677–684 18278832

[B16] Hampson S. E. Glasgow R. E. Zeiss A. M. (1996). Coping with osteoarthritis by older adults. Arthritis Care Res. 9, 133–141 10.1002/1529-0131(199604)9:2<133::AID-ANR1790090210>3.0.CO;2-9 8970272

[B17] Hasenbring M. I. Plaas H. Fischbein B. Willburger R. (2006). The relationship between activity and pain in patients 6 months after lumbar disc surgery: do pain-related coping modes act as moderator variables? Eur. J. Pain 10, 701–709 10.1016/j.ejpain.2005.11.004 16426878

[B18] Hasenbring M. I. Verbunt J. A. (2010). Fear-avoidance and endurance-related responses to pain: new models of behavior and their consequences for clinical practice. Clin. J. Pain 26, 747–753 10.1097/AJP.0b013e3181e104f2 20664333

[B19] Hill C. L. Parsons J. Taylor A. Leach G. (1999). Health related quality of life in a population sample with arthritis. J. Rheumatol. 26, 2029–2035 10493687

[B20] Hopman-Rock M. Kraaimaat F. W. Odding E. Bijlsma J. W. (1998). Coping with pain in the hip or knee in relation to physical disability in community-living elderly people. Arthritis Care Res. 11, 243–252 10.1002/art.1790110405 9791323

[B21] Huber P. (1967). “The behavior of the maximum likelihood estimates under nonstandard conditions,” in Proceedings of the Fifth Berkeley Symposium on Mathematical Statistics and Probability, Vol. 1 (Berkeley: University of California Press), 221–233

[B22] Huijnen I. P. J. Verbunt J. A. Peters M. L. Delespaul P. Kindermans H. P. J. Roelofs J. Goossens M. Seelen H. A. M. (2010). Do depression and pain intensity interfere with physical activity in daily life in patients with chronic low back pain? Pain 150, 161–166 10.1016/j.pain.2010.04.021 20457489

[B23] Huijnen I. P. J. Verbunt J. A. Peters M. L. Smeets R. J. E. M. Kindermans H. P. J. Roelofs J. Goossens M. Seelen H. A. M. (2011). Differences in activity-related behaviour among patients with chronic low back pain. Eur. J. Pain 15, 748–755 10.1016/j.ejpain.2010.11.015 21195646

[B24] Huijnen I. P. J. Verbunt J. A. Roelofs J. Goossens M. Peters M. (2009). The disabling role of fluctuations in physical activity in patients with chronic low back pain. Eur. J. Pain 13, 1076–1079 10.1016/S1090-3801(09)60936-8 19181547

[B25] Jensen M. P. (1991). Coping with chronic pain: a critical review of the literature. Pain 47, 249–283 10.1016/0304-3959(91)90216-K 1784498

[B26] Jensen M. P. Turner J. A. Romano J. M. Strom S. E. (1995). The chronic pain coping inventory: development and preliminary validation. Pain 60, 203–216 10.1016/0304-3959(94)00118-X 7784106

[B27] Karsdorp P. Vlaeyen J. W. S. (2009). Active avoidance but not activity pacing is associated with disability in fibromyalgia. Pain 147, 29–35 10.1016/j.pain.2009.10.002 19716234

[B28] Keller S. Bann C. M. Dodd S. L. Schein J. Mendoza T. R. Cleeland C. S. (2004). Validity of the brief pain inventory for use in documenting the outcomes of patients with noncancer pain. Clin. J. Pain 20, 309–318 10.1097/00002508-200409000-00005 15322437

[B29] Kellgren J. Lawrence J. (1957). Radiological assessment of osteoarthrosis. Ann. Rheum. Dis. 16, 494–501 10.1136/ard.16.4.511-c 13498604 PMC1006995

[B30] Kindermans H. P. J. (2011). “Being” in pain: the role of self-discrepancies in the emotional experience and activity patterns of patients with chronic low back pain. Pain 152, 403–409 10.1016/j.pain.2010.11.009 21216100

[B31] Kindermans H. P. J. Roelofs J. Goossens M. E. J. B. Huijnen I. P. J. Verbunt J. A. Vlaeyen J. W. S. (2011). Activity patterns in chronic pain: underlying dimensions and associations with disability and depressed mood. J. Pain 12, 1049–1058 10.1111/j.1526-4637.2011.01156.x 21704568

[B32] Lazarus R. S. Folkman S. (1984). Stress, Appraisal, and Coping. New York: Springer

[B33] Leeuw M. (2007). The fear-avoidance model of musculoskeletal pain: current state of scientific evidence. J. Behav. Med. 30, 77–94 10.1007/s10865-006-9085-0 17180640

[B34] McCracken L. Samuel V. (2007). The role of avoidance, pacing, and other activity patterns in chronic pain. Pain 130, 119–125 10.1016/j.pain.2006.11.016 17240065

[B35] Mendoza T. R. Wang X. S. Cleeland C. S. Morrissey M. Johnson B. A. Wendt J. K. Huber S. L. (1999). The rapid assessment of fatigue severity in cancer patients: use of the brief fatigue inventory. Cancer 85, 1186–1196 10.1002/(SICI)1097-0142(19990301)85:5<1186::AID-CNCR24>3.0.CO;2-N 10091805

[B36] Murphy S. L. (2009). Review of physical activity measurement using accelerometers in older adults: considerations for research design and conduct. Prev. Med. 48, 108–114 10.1016/j.ypmed.2008.12.001 19111780 PMC10071821

[B37] Murphy S. L. Clauw D. J. (2010). Activity pacing: what are we measuring and how does it relate to treatment? Pain 149, 582–583 10.1016/j.pain.2010.03.031 20381246

[B38] Murphy S. L. Lyden A. K. Clary M. Geisser M. Yung R. Welch K. Clauw D. J. Williams D. A. (2011). Activity pacing for osteoarthritis symptom management: study design and methodology of a randomized trial testing a tailored clinical approach using accelerometers for veterans and non-veterans. BMC Musculoskelet. Disord. 12, 177 10.1186/1471-2474-12-177 21810253 PMC3162944

[B39] Murphy S. L. Smith D. M. Alexander N. B. (2008a). Measuring activity pacing in women with lower-extremity osteoarthritis: a pilot study. Am. J. Occup. Ther. 62, 329–334 10.5014/ajot.62.3.329 18557009 PMC3039298

[B40] Murphy S. L. Smith D. M. Clauw D. J. Alexander N. B. (2008b). The impact of momentary pain and fatigue on physical activity in women with osteoarthritis. Arthritis Rheum. 59, 849–856 10.1002/art.23710 18512720 PMC3046423

[B41] Murphy S. L. Smith D. M. Lyden A. K. (2012). Type of activity pacing instruction affects physical activity variability in adults with symptomatic knee or hip osteoarthritis. J. Phys. Act. Health 9, 360–366 21934157 10.1123/jpah.9.3.360 PMC3637666

[B42] Nielson W. R. Jensen M. P. (2004). Relationship between changes in coping and treatment outcome in patients with fibromyalgia syndrome. Pain 109, 233–241 10.1016/j.pain.2004.01.002 15157683

[B43] Nielson W. R. Jensen M. P. Hill M. L. (2001). An activity pacing scale for the chronic pain coping inventory: development in a sample of patients with fibromyalgia syndrome. Pain 89, 111–115 10.1016/S0304-3959(00)00351-1 11166466

[B44] Norton P. J. Asmundson G. J. G. (2003). Amending the fear-avoidance model of chronic pain: what is the role of physiological arousal? Behav. Ther. 34, 17–30 10.1016/S0005-7894(03)80019-9

[B45] Patterson S. M. Krantz D. S. Montgomery L. C. Deuster P. A. Hedges S. M. Nebel L. E. (1993). Automated physical activity monitoring: validation and comparison with physiological and self-report measures. Psychophysiology 30, 296–305 10.1111/j.1469-8986.1993.tb03356.x 8497559

[B46] Podsiadlo D. Richardson S. (1991). The timed “Up & Go”: a test of basic functional mobility for frail elderly persons. J. Am. Geriatr. Soc. 39, 142–148 1991946 10.1111/j.1532-5415.1991.tb01616.x

[B47] Power J. D. Perruccio A. V. Badley E. M. (2005). Pain as a mediator of sleep problems in arthritis and other chronic conditions. Arthritis Care Res. 53, 911–919 10.1002/art.21584 16342098

[B48] Radloff L. (1977). The CES-D scale: a self-report depression scale for research in the general population. Appl. Psychol. Meas. 1, 385–401 10.1177/014662167700100306

[B49] Spielberger C. D. Gorsuch R. L. Lushene R. E. (1970). Manual for the State-Trait Anxiety Inventory. Palo Alto, CA: Consulting Psychologists Press

[B50] Steultjens M. P. (2001). Coping, pain, and disability in osteoarthritis: a longitudinal study. J. Rheumatol. 28, 1068–1072 11361191

[B51] Swartz A. M. Strath S. J. Bassett D. R. O’Brien W. L. King G. A. Ainsworth B. E. (2000). Estimation of energy expenditure using CSA accelerometers at hip and wrist sites. Med. Sci. Sports Exerc. 32, S450–S456 10.1097/00005768-200009001-00003 10993414

[B52] Tan G. Jensen M. P. Robinson-Whelen S. Thornby J. I. Monga T. N. (2001). Coping with chronic pain: a comparison of two measures. Pain 90, 127–133 10.1016/S0304-3959(00)00395-X 11166978

[B53] Tan G. Teo I. Anderson K. O. Jensen M. P. (2011). Adaptive versus maladaptive coping and beliefs and their relation to chronic pain adjustment. Clin. J. Pain 27, 769–774 10.1097/AJP.0b013e31821d8f5a 21593665

[B54] van Baar M. E. Dekker J. Lemmens J. A. Oostendorp R. A. Bijlsma J. W. (1998). Pain and disability in patients with osteoarthritis of hip or knee: the relationship with articular, kinesiological, and psychological characteristics. J. Rheumatol. 25, 125–133 [See comment]. 9458215

[B55] Vendrig A. A. Lousberg R. (1997). Within-person relationships among pain intensity, mood and physical activity in chronic pain: a naturalistic approach. Pain 73, 71–76 10.1016/S0304-3959(97)00075-4 9414058

[B56] Vlaeyen J. W. Linton S. J. (2000). Fear-avoidance and its consequences in chronic musculoskeletal pain: a state of the art. Pain 85, 317–332 10.1016/S0304-3959(99)00242-0 10781906

[B57] Ware J. Kosinski M. Keller S. (1996). A 12-item short-form health survey: construction of scales and preliminary tests of reliability and validity. Med. Care 34, 220–233 10.1097/00005650-199603000-00003 8628042

[B58] West S. G. Finch J. F. Curran P. J. (1995). Structural Equation Models with Non-Normal Variables: Problems and Remedies. Newbury Park, CA: Sage

[B59] Westerterp K. R. (1999). Physical activity assessment with accelerometers. Int. J. Obes. Relat. Metab. Disord. 23(Suppl. 3), S45–S49 10.1038/sj.ijo.0800810 10368002

[B60] White H. (1980). A heteroskedasticity-consistent covariance matrix estimator and a direct test for heteroskedasticity. Econometrica 48, 817–838 10.2307/1912934

[B61] Wolfe F. (1999). Determinants of WOMAC function, pain and stiffness scores: evidence for the role of low back pain, symptom counts, fatigue and depression in osteoarthritis, rheumatoid arthritis and fibromyalgia. Rheumatology 38, 355–361 10.1093/rheumatology/38.4.355 10378714

[B62] Wolfe F. Hawley D. J. Wilson K. (1996). The prevalence and meaning of fatigue in rheumatic disease. J. Rheumatol. 23, 1407–1417 8856621

